# Action observation training to improve motor function recovery: a systematic review

**DOI:** 10.1186/s40945-015-0013-x

**Published:** 2015-12-02

**Authors:** Elisabetta Sarasso, Mariano Gemma, Federica Agosta, Massimo Filippi, Roberto Gatti

**Affiliations:** 1 Rehabilitation Department, San Raffaele Scientific Institute, Milan, Italy; 2 Neuroimaging Research Unit, INSPE, Division of Neuroscience, San Raffaele Scientific Institute, Vita-Salute San Raffaele University, Milan, Italy; 3 School of Physiotherapy, Vita-Salute San Raffaele University, Milan, Italy

**Keywords:** Action observation, Motor function recovery, Systematic review

## Abstract

Following the discovery of Mirror Neuron System (MNS), Action Observation Training (AOT) has become an emerging rehabilitation tool to improve motor functions both in neurologic and orthopedic pathologies.

The aim of this study is to present the state of the art on the use of AOT in experimental studies to improve motor function recovery in any disease.

The research was performed in PubMed, PEDro, Embase, CINAHL and Cochrane Central Register of Controlled Trials (last search July 2015). Randomized controlled trials (RCTs) that analyse efficacy of AOT for recovery of motor functions, regardless of the kind of disease, were retrieved. The validity of the included studies was assessed using the Cochrane Collaboration tool for evaluating risk of bias.

Twenty RCTs were eligible. Four studies showed AOT efficacy in improving upper limb functional recovery in participants with chronic stroke, two studies in sub-acute ones and one in acute ones. Six articles suggested its effectiveness on walking performance in chronic stroke individuals, and three of them also suggested an efficacy in improving balance. The use of AOT was also recommended in individuals with Parkinson’s disease to improve autonomy in activities of daily living, to improve spontaneous movement rate of self-paced finger movements and to reduce freezing of gait. Other two studies also indicated that AOT improves upper limb motor function in children with cerebral palsy. The last two studies, showed the efficacy of AOT in improving motor recovery in postsurgical orthopedic participants. Overall methodological quality of the considered studies was medium.

The majority of analyzed studies suggest the efficacy of AOT, in addition to conventional physiotherapy, to improve motor function recovery in individuals with neurological and orthopedic diseases. However, the application of AOT is very heterogeneous in terms of diseases and outcome measures assessed, which makes it difficult to reach, to date, any conclusion that might influence clinical practice.

**Electronic supplementary material:**

The online version of this article (doi:10.1186/s40945-015-0013-x) contains supplementary material, which is available to authorized users.

## Background

Mirror Neurons (MN) were described for the first time in the nineties by a group of researchers at the University of Parma, and localized in the ventral premotor cortex (F5 area) of macaques. [1] In this region, two types of neurons were identified: the canonical neurons, which respond during goal directed hand movement, and the visuo-motor mirror neurons, which are activated both when the monkey performs a particular motor gesture directed toward an object and when this action is seen without executing. The existence of MN in humans has been confirmed by studies performed with Transcranial Magnetic Stimulation (TMS) [2] and non-invasive neuroimaging techniques [3] that demonstrated the presence of classes of neurons that are compatible with those observed in macaques. In humans, MN have also been described in the rostral part of the inferior parietal lobule (IPL), whose properties appear to be similar to those of neurons in the premotor cortex. These two areas are connected together and form a network which is a part of the fronto-parietal circuit that organizes actions [4, 5]. MN of humans also play an important role in understanding the intentions of other actions. Functional MRI (fMRI) studies indeed confirmed the same activation of MN both when the intent of the subject is easily understandable and when it is ambiguous [6].

The discovery that MN are involved in motor learning [7] has allowed the development of a new rehabilitation approach, called Action Observation Training (AOT), during which the patient is asked to carefully observe actions presented through a video-clip or performed by an operator, in order to try and imitate them after the observation. The purpose of AOT in the rehabilitation of individuals with lesions of the central nervous system (CNS) is to provide a tool to recover damaged cerebral networks [8] and take advantage to rebuild motor function despite impairments, as an alternative or complement to physiotherapy [9]. Several studies [10, 11] confirmed the hypothesis that the imitation of observed gestures lead to a reorganization of the primary motor cortex, contributing to the formation of motor memory of the observed action, physiological process underlying motor learning. The clinical relevance is easy to understand: when a patient is unable to perform movements because of neural damage or pain or imposed immobility, AOT offers the possibility to activate specific areas of the cerebral cortex, reinforcing intact cortical networks and facilitating the activation of the damaged ones, preventing changes in cortical reorganization that occur after inactivity and disuse [12]. Based on these findings, in the last ten years several studies on the clinical use of AOT have been published.

## Review

### Objectives

The aim of this study is to present a systematic review on the use of AOT in experimental studies to improve motor function recovery in any disease. It was decided to investigate the modality of application and the posology of this technique, the diseases on which it was applied, the objectives and the outcome measures used to assess its efficacy.

## Materials and methods

### Inclusion and exclusion criteria

Randomized controlled trials (RCTs) that focused on the effects of a period of AOT on motor rehabilitation were included. There was no restriction on disease, impairment and disability of the participants. The following selection criteria (PICO) were used: a randomized controlled trial design, a patient population including any kind of disease, a rehabilitative intervention focused on AOT, outcomes of motor function recovery. All the articles had to be available in English and full-text.

### Search strategy

Pubmed (from 1950), PEDro (from 1929), Embase (from 1980), CINAHL (from 1982) and the Cochrane Central Register of Controlled Trials (from 1929) databases were electronically searched until July 2015. Three key terms – action observation, rehabilitation, and motor function - were used to generate a list of search terms, which were combined into a search strategy adapted to each database: (action OR motor OR movement) AND observation AND (training OR treatment OR therapy OR physical training OR movement execution OR rehabilitation OR neurorehabilitation) AND ((motor AND (function OR recovery OR learning OR activity OR ability)) OR (functional recovery). The extended version is available in Additional file 1.

### Study selection

**Fig. 1:**
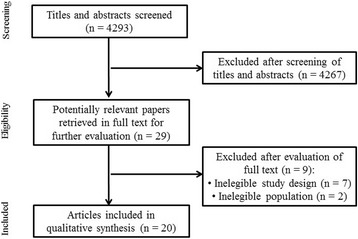
Flowchart of study selection

Among the articles found by the search (see flowchart in Fig. 1), 29 were selected according to the titles and abstracts by two independent reviewers (ES, MG). Reference lists of identified studies and published reviews were manually checked for additional RCTs. References retrieved by the electronical search were compared for duplicate entries and were manually cross-checked. Eligible papers were gathered in full-text, independently screened by the same reviewers. A third reviewer (RG) facilitated decision-making when there was disagreement.

### Assessment of risk of bias in included studies

The risk of bias of the included studies was independently assessed by two review authors (ES and MG) using the Cochrane Collaboration’s “Risk of bias” tool [13]. The assessment was achieved by assigning a judgment of ‘low risk’ of bias when bias was considered unlikely to have altered the results, ‘high risk’ of bias when the potential for bias weakened confidence in the results, or ‘unclear risk’ when there was some doubt about the effect of bias on the results. The following topics were assessed: description of randomization, allocation concealment, blinding, completeness of outcome data, and selective reporting [13]. Considering the nature of the intervention, blinding of the physiotherapists administering the AOT was impractical, so only outcome assessor and participant blinding was considered.

## Results

Two of the 29 articles were excluded because they were study protocols [14, 15], five because they were not RCTs [16–20] and two because they were about AOT in healthy subjects (young or elderly) [21, 22]. The letter Buccino et al. 2011 was included despite not being a full RCT research paper, since as authors of the letter we can confirm it fulfilled the necessary requirements to be included in our analysis. As a result of the screening, 20 articles were included in the review (see Table 1).

**Table 1:** Included articles ordered by year of publication

Title	Authors	Journal	Year
A mirror therapy-based action observation protocol to improve motor learning after stroke.	Harmsen WJ, Bussmann JB, Selles RW, Hurkmans HL, Ribbers GM.	Neurorehabil Neural Repair	2015
The effects of action observation gait training on the static balance and walking ability of stroke patients.	Park EC, Hwangbo G.	J Phys Ther Sci	2015
Effects of purposeful action observation on kinematic patterns of upper extremity in individuals with hemiplegia.	Kim E, Kim K.	J Phys Ther Sci	2015
The effect of action observation training on knee joint function and gait ability in total knee replacement patients.	Park SD, Song HS, Kim JY.	J Exerc Rehabil	2014
Action observation therapy in the sub-acute phase promotes dexterity recovery in right-hemisphere stroke patients.	Sale P, Ceravolo MG, Franceschini M.	Biomed Res Int	2014
Clinical feasibility of action observation training for walking function of patients with post-stroke hemiparesis: a randomized controlled trial.	Park HR, Kim JM, Lee MK, Oh DW.	Clin Rehabil	2014
Action observation training for functional activities after stroke: A pilot randomized controlled trial.	Kim JH, Lee BH.	NeuroRehabilitation	2013
The effects of action observational training on walking ability in chronic stroke patients: a double-blind randomized controlled trial.	Bang DH, Shin WS, Kim SY, Choi JD.	Clin Rehabil	2013
The effects of additional action observational training for functional electrical stimulation treatment on weight bearing, stability and gait velocity of hemiplegic patients.	Park CS, Kang KY.	J Phys Ther Sci	2013
Reduction of bradykinesia of finger movements by a single session of action observation in Parkinson disease.	Pelosin E, Bove M, Ruggeri P, Avanzino L, Abbruzzese G.	Neurorehabil Neural Repair	2013
Drinking behavior training for stroke patients using action observation and practice of upper limb function.	Lee D, Roh H, Park J, Lee S, Han S.	J Phys Ther Sci	2013
Observation-to-imitate plus practice could add little to physical therapy benefits within 31 days of stroke: translational randomized controlled trial.	Cowles T, Clark A, Mares K, Peryer G, Stuck R, Pomeroy V.	Neurorehabil Neural Repair	2013
Randomized trial of observation and execution of upper extremity actions versus action alone in children with unilateral cerebral palsy.	Sgandurra G, Ferrari A, Cossu G, Guzzetta A, Fogassi L, Cioni G.	Neurorehabil Neural Repair	2013
Improving upper limb motor functions through action observation treatment: a pilot study in children with cerebral palsy.	Buccino G, Arisi D, Gough P, Aprile D, Ferri C, Serotti L, Tiberti A, Fazzi E.	Dev Med Child Neurol	2012
Clinical relevance of action observation in upper-limb stroke rehabilitation: a possible role in recovery of functional dexterity. A randomized clinical trial.	Franceschini M, Ceravolo MG, Agosti M, Cavallini P, Bonassi S, Dall’Armi V, Massucci M, Schifini F, Sale P.	Neurorehabil Neural Repair	2012
Clinical feasibility of action observation based on mirror neuron system in walking performance on post stroke patients.	Kim JS, Kim K.	J Phys Ther Sci	2012
Action observation treatment improves autonomy in daily activities in Parkinson’s disease patients: results from a pilot study.	Buccino G, Gatti R, Giusti MC, Negrotti A, Rossi A, Calzetti S, Cappa SF.	Mov Disord	2011
Action observation treatment improves recovery of postsurgical orthopedic patients: evidence for a top-down effect?	Bellelli G, Buccino G, Bernardini B, Padovani A, Trabucchi M.	Arch Phys Med Rehabil	2010
Action observation improves freezing of gait in patients with Parkinson’s disease.	Pelosin E, Avanzino L, Bove M, Stramesi P, Nieuwboer A, Abbruzzese G.	Neurorehabil Neural Repair	2010
Action observation has a positive impact on rehabilitation of motor deficits after stroke.	Ertelt D, Small S, Solodkin A, Dettmers C, McNamara A, Binkofski F, Buccino G.	Neuroimage	2007

### Characteristics of included studies

The characteristics of included studies are summarized in the Table 2 and extensively reported in the Additional file 2.

**Table 2:** Summary of included studies

Study	Participants	Intervention	Outcome measures
Harmsen W. J. et al. [24]	37 participants in the chronic stage after stroke Exp: n = 18 (9 F) Age: 57 (SD 10.4) Con: n = 19 (6 F) Age: 60 (SD 8.8)	Exp: observation of the upper-arm reaching movements similar to what patients would see in the mirror during mirror therapy (3 + 1 + 1 + 1 min) + execution of reaching movements (30 + 20 + 20 reps) Con: observation of a slideshow with static photographs of landscapes (3 + 1 + 1 + 1 min) + execution of reaching movements (30 + 20 + 20 reps)	Upper limb Kinematics: movement time and acceleration during a reaching movement.
Park E.C. et al. [33]	40 chronic stroke individuals Exp: n = 20 (10 F) Age = 51.15 (SD 14.81) Con: n = 20 (9 F) Age = 48.65 (SD 12.81)	Both: 30 min PT, 5/wk x 8wk Exp: observation of 3-minute videos of walking (3 videos) + walking training for 20 minutes (30 min, 5/wk x 8wk) Con: observation of 3-minute landscapes videos (3 videos) + walking training for 20 minutes (30 min, 5/wk x 8wk)	Balance ability = analysis system using biofeedback (LOS, SS, SA) Gait ability = TUG, 10MWT
Kim E. et al. [25]	12 stroke individuals Exp: n = 6 Con: n = 6	Exp: traditional occupational treatment + purposeful action observation training program (30 min, 5/wk x 6wk) Con: traditional occupational treatment + topological treatment in which they performed purposeful action observation program assignments without actually observing the purposeful actions (30 min, 5/wk x 6wk)	Upper limb Kinematics: average velocity, trajectory ratio, motion angle
Sale et al. [28]	67 sub-acute stroke individuals (26 F) Age = 66.5 (SD 12.7)	Exp: observation of 3-minute videos of manual tasks (3 videos) + execution of observed movement for 2 minutes (3 times) (2 x 15 min, 5/wk x 4wk) Con: observation of 5 static images displaying objects (3 minutes) + execution of same movement as exp group for 2 minutes (3 times) (2 x 15 min, 5/wk x 4wk)	Manual dexterity: BBT Motor impairment: FMA Follow up = 4–5 mth
Park H.R. et al. [34]	21 chronic stroke individuals Exp: n = 11 (3 F), Age = 55.9 (SD 9.1) Con: n = 10 (3 F) Age = 54.8 (SD 12.22)	Exp: observation of 10 minutes of video demonstrating four tasks for functional walking + 20 min walking training (30 min, 3/wk x 4wk) Con: 10 minutes of different landscape images + 20 min walking training (30 min, 3/wk x 4wk)	Gait ability = 10 MWT, F8WT, DGI, Gait symmetry scores
Kim J.H. et al. [31]	30 chronic stroke individuals Exp AOT: n = 9 (2 F), Age = 55.3 (SD 12.1) Exp Mi: n = 9 (3 F) Age = 54.8 (SD 8.8) Con: n = 9 (2 F) Age = 59.8 (SD 8.9)	Exp AOT: observation of 20 minutes-video followed by physical training (10 minutes) (30 min, 5/wk x 4wk) Exp Mi: 20 minutes of Mi followed by physical training for 10 minutes(30 min, 5/wk x 4wk) Con: Physical training (30 min, 5/wk x 4wk)	Gait ability = TUG, WAQ, FAC Gait kinematics = Spatiotemporal gait parameters Balance = FRT
Bang et al. [30]	30 chronic stroke individuals Exp: n = 15 (6 F), Age = 64.1 (SD 6.35) Con: n = 15 (7 F) Age = 58.9 (SD 6.03)	Exp: treadmill training (30 min) after watching the treadmill video (9 min) (40 min, 5/wk x 4wk) Con: treadmill training (30 min) after watching the nature video (9 min). (40 min, 5/wk x 4wk)	Gait ability = TUG, 10MWT, 6MWT Gait kinematics = Knee angle in swing phase during walking
Park C.S. et al. [35]	20 chronic stroke individuals Exp: n = 10 (4 F) Con: n = 10 (5 F)	Both: functional electrical stimulation treatment. Exp = observation of a video on gait for 15 minutes. The video showed walking on flat ground, slopes and stairs. (15 min, 5/wk x 4wk)	Balance ability: Weight Distribution, Stability Index Gait kinematics: Gait Velocity
Lee D. et al. [26]	33 chronic stroke individuals Action observation group: n = 8 (3 F), Age: 63 (SD 3.7) Action practice group: n = 9 (4 F) Age: 62 (SD 1.5) Combination group: n = 9 (4 F) Age: 61 (SD 2.3) Control group: n = 7 (4 F) Age: 60 (SD 5.9)	The action observation group watched a video of the task (10 minutes), the action practice group performed the action (10 minutes), the combined action observation-action practice group watched the video of the task (5 minutes) and practiced the action (5 minutes), and the control group did not perform either action observation or action practice.	Upper-limb functional dexterity: number of times the full drinking action was performed in one minute Follow up = 1 wk
Cowles et al. [29]	29 acute stroke individuals Exp: n = 15 (7 F), Age = 78.8 (SD 8.1) Con: n = 14 (5 F) Age = 75.6 (SD 12.4)	Exp: 3 x 8 min imitation of therapist performing functional activities with upper limb (2 x 30 min, 15 consecutive working days) Con: no therapy in addition to conventional physiotherapy	Ability to voluntarily contract paretic muscle = MI Functional use of upper limb = ARAT
Franceschini et al. [27]	102 sub-acute stroke individuals Exp: n = 53 (20 F) Age = 67.0 (SD 12.4) Con: n = 49 (21 F) Age = 65.7 (SD 11.9)	Exp: observation of 3-minute videos of manual tasks (3 videos) + execution of observed movement for 2 minutes (3 times) (2 x 15 min, 5/wk x 4wk) Con: observation of 5 static images displaying objects (3 minutes) + execution of same movement as exp group for 2 minutes (3 times) (2 x 15 min, 5/wk x 4wk)	Upper-limb functional dexterity: FAT, BBT, FIMM Motor impairment: FMA Follow up = 4–5 mth
Kim JS et al. [32]	30 chronic stroke individuals Exp: n = 15, Age = 64.1 (SD 8.3) Con: n = 15 Age = 65.5 (7.7)	Both: 30 min PT Exp: observation of 2-minute videos of walking (5 videos) + walking training for 10 minutes Con: 10 minutes of video in which they were taken through a progressive relaxation program (stretching)	Gait kinematics: Spatiotemporal gait parameters (including walking speed)
Ertelt et al. [23]	15 chronic stroke individuals Exp: n = 7 (2 F) Age = 57.16 (SD 8.73) Con: n = 8 (2 F) Age = 55.40 (SD 10.77)	(90 min x 18 consecutive working days) Exp: observation of 6-minute videos of daily life hand and arm actions (3 videos) + execution of observed movement for 6 minutes (3 times) Con: observation of sequences of geometric symbols and letters + execution of same movement as exp group for 6 minutes (3 times)	Upper-limb functional dexterity: WMFT, FAT Subjective scale: SIS Follow up = 8 wk
Pelosin et al. [38]	38 individuals with PD (21 F) Age = 67.4 (SD 7.4) 14 matched healthy controls (7 F)	VIDEO group: observation of a 6-minute video clip showing repetitive finger movements paced at 3 Hz ACOUSTIC group: listening to an acoustic cue paced at 3 Hz for 6 minutes. 8 participants with PD were recruited for a sham intervention, watching a 6-minute video representing a static hand.	Spontaneous movement rate (SMR) of self-paced finger movements. Follow up = 2 days
Buccino et al. [36]	15 PD individuals Exp: n = 7 Age = 68 (2 F) (min-max: 59–80) Con: n = 8 Age = 73.5 (3 F) (min-max: 67.5–76.5)	Exp: observation and subsequently execution of different daily actions presented through video clips Con: observation of video clips with no motor content and subsequently performance of the same actions as exp group	Autonomy in ADL: UPDRS, FIM
Pelosin et al. [37]	18 individuals with PD (8 F) Exp: n = 9 Age = 68.8 (SD 4.1) Con: n = 9 Age = 70.2 (SD 6.8)	Exp: observation of 6-minute videos of walking tasks (4 videos) + execution of the same motor training (36 minutes) (60 min, 3/wk x 4wk) Con: observation of landscape videos + execution of same exercises in the exact order and for the same amount of time than AOT group (60 min, 3/wk x 4wk)	FoG frequency and severity: FoG-Q, FoG-diary Disease severity: H&Y Motor impairment: UPDRSIII Gait ability = TUG, 10MWT Balance ability: BBS, Tinetti score Quality of life: PDQ-39 Follow up = 4 wk
Sgandurra et al. [40]	24 cerebral palsy children Exp: n = 12 (4 F) Age 9.48 (SD 2.12) Con: n = 12 (4 F) Age 9.94 (SD 2.77)	Exp: observation of 3 minutes-videos representing upper limb actions + execution of the same motor tasks (3 minutes) (60 min x 15 consecutive working days) Con: observation of computer games + execution of the same motor tasks as exp group (60 min x 15 consecutive working days)	Upper limb function = AHA, MUUL Manual ability = ABILHAND-Kids questionnaire Follow up = 1-8-24 wk
Buccino et al. [39]	24 cerebral palsy children Exp: n = 8 (4 F) Con: n = 7 (2 F)	Exp: observation of 9–12 minutes-videos representing upper limb actions + execution of the same motor tasks (6–8 minutes) (5/wk x 3wk) Con: observation of history, geography or science videos (9–12 minutes) + execution of the same motor tasks as exp group (6–8 minutes) (5/wk x 3wk)	Upper limb function = MaS
Park S.D. et al. [34]	18 individuals with TKR Exp: n = 9 Age = 72.67 (SD 12.25) Con: n = 9 Age = 70.56 (SD 10.98)	Both: 30 minutes of gait exercise and treadmill Exp: observation of a 10 \minutes-video clip showing daily actions + execution (30 minutes) of the same actions (3/wk x 3wk) Con: execution of the same actions as patients in the AOT group (30 minutes) (3/wk x 3wk)	Scale for Osteoarthritis = WOMAC (including pain, stiffness and function) Gait ability = TUG
Bellelli et al. [41]	60 individuals (hip fractures or hip or knee replacement) Exp: n = 30 (21 F) Age = 71.9 (SD 8.4) Con: n = 30 (16 F) Age = 71.8 (SD 6.9)	Both: 60 min conventional post-orthopedic rehabilitation Exp: observation of 8 minutes-video clips showing daily actions (3 videos) + execution of the same actions (6/wk x 3wk) Con: observation geographic documentary + execution of the same actions as patients in the AOT group (6/wk x 3wk)	Functional ability: FIM, FIMM Gait ability: Tinetti, types of walking aids Follow up = 1-2-3 wk

*Exp* experimental group, *Con* control group, *F* female, *wk* week, *reps* repetitions, *PT* physical therapy, *LOS* limit of stability, *SS* sway speed, *SA* sway area, *TUG* timed up and go, *10MWT* 10-metre walk test, *BBT* box and block test, *FMA* Fugl-Meyer Assessment, *F8WT* figure-of-8 walk test, *DGI* dynamic gait index, *AOT* action observation training, *Mi* motor imagery, *WAQ* walking ability questionnaire, *FRT* functional reach test, *FAC* functional ambulation categories, *6MWT* 6-minute walking test, *MI* motricity index, *ARAT* action research arm test, *FIMM* functional independence measure motor score, *WMFT* wolf motor funtion test, *SIS* stroke impact scale, *PD* Parkinson’s disease, *ADL* activity of daily living, *FoG-Q* freezing of gait questionnaire, *H&Y* Hoen and Year scale, *UPDRS* unified Parkinson’s disease rating scale, *BBS* Berg balance scale, *PDQ-39* Parkinson’s disease questionnaire 39, *AHA* assisting hand assessment, *MUUL* Melbourne assessment of unilateral upper limb function, *MaS* Melbourne assessment scale, *TKR* total knee replacement, *WOMAC* Western Ontario and Mc-Master Universities osteoarthritis index

### Participants

Seven studies involved stroke individuals with upper limb impairment: 97 chronic stroke subjects in four studies [23–26], 169 sub-acute stroke participants in two studies [27, 28], and 29 acute stroke individuals in one study [29]. Six articles [30–35] investigated a population of 171 chronic stroke subjects with walking deficits, and three [33–35] of them also balance deficits (*N* = 90). The use of AOT was also explored in three samples of 15 [36], 18 [37] and 38 [38] participants with Parkinson’s disease. Other two studies analyzed a population of 48 children with cerebral palsy [39, 40] with upper limb motor impairment. Finally, two studies [41, 42] investigated the effect of AOT in 78 postsurgical orthopedic individuals.

#### Intervention

In the experimental (AOT) group, 30 % of studies [25, 32, 33, 35, 41, 42] combined AOT to standard rehabilitation. The majority of studies resulted in an equal treatment time between the experimental and control groups. All the studies administered AOT through videos with the exception of one [29] in which subjects had to imitate actions performed by a physiotherapist. The characteristics of the intervention expressed as mean values (range) were: 12.4 min of AOT for each session (5–30); 7.4 min for each video; 16.9 min of observed actions performance (5–36); 6 sessions a week (3–10); total duration of treatment = 16.2 days (1–40). In all studies, with the except of three [29, 31, 32], the control group performed the same actions of the experimental group for the same amount of time. The only difference was that the control group watched videos without motor contents (landscapes, documentaries, geometric shapes, etc.), with the except of four studies [29, 31, 32, 42]: in one [32] of them the videos showed stretching exercises, in the other studies [29, 31, 35, 42] the control group did not see any video. Only one study [26] compared the effects of 10 min of “standard” AOT (5 min video, 5 min repetition) relative to 10 min of observation or 10 min of imitation alone. Finally, a study [38] investigated the different effects of a single-session of action observation without execution, relative to both a single-session of listening an acoustic cue and a single-session of static video, in improving spontaneous movement rate of self-paced finger movements in participants with Parkinson’s disease.

#### Outcome measures

In keeping with the heterogeneous patient population included into the studies, also the outcomes used were very mixed. All outcomes are listed in Table 2 and in Additional file 2. Only RCTs including individuals with stroke showed a consistency in the use of outcome measures. Indeed, two studies [27, 28] used the Box and Block test and two [23, 27] the Frenchay Arm test to assess upper limb functional dexterity in sub-acute/chronic stroke participants, and three studies [30, 31, 33] used the Time Up and Go test (TUG) and three [30, 33, 34] the 10 m walking test (10MWT) to assess the walking ability in chronic subjects. Overall, in the 20 detected articles, 37 outcomes were administered.

#### Quality

**Fig. 2:**
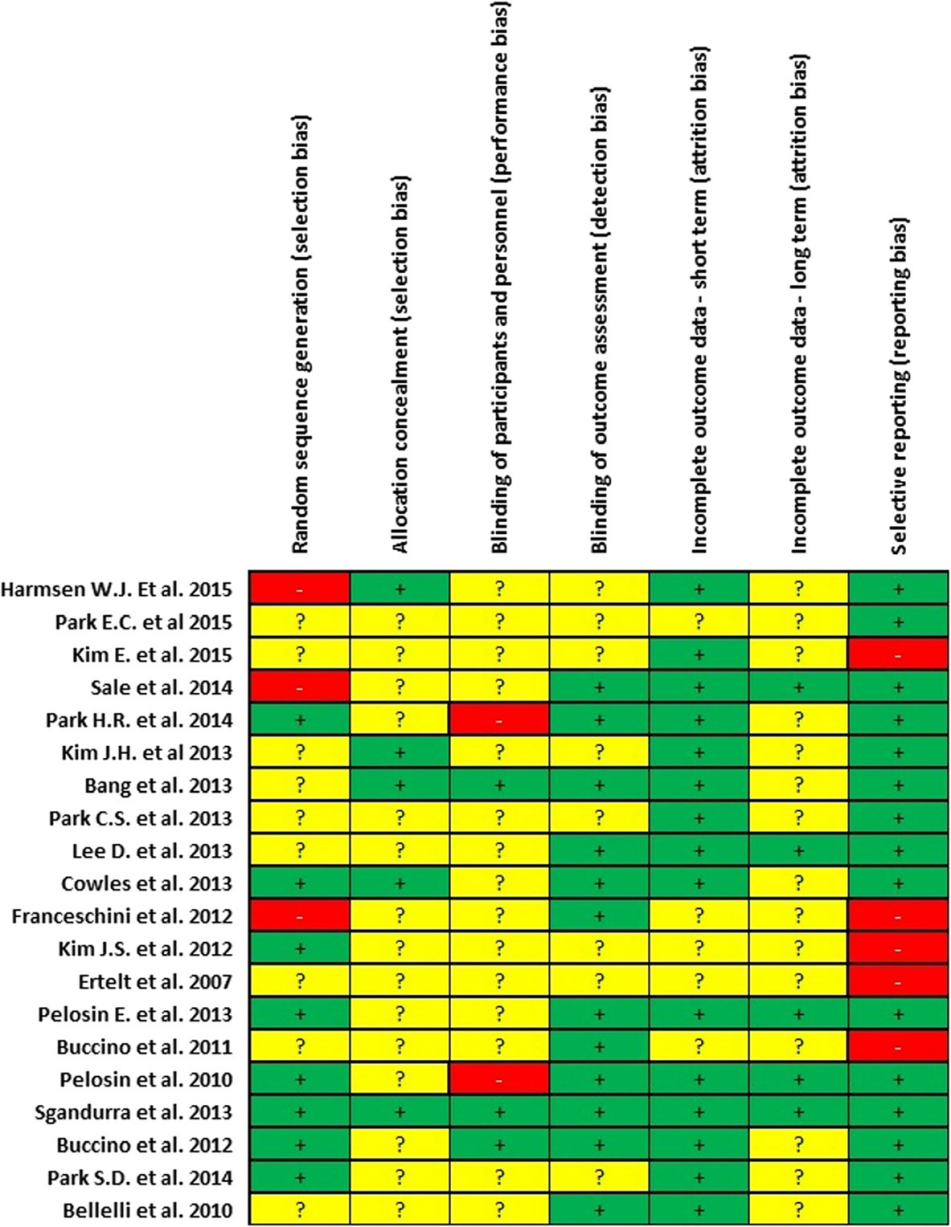
Overview of the risk of bias according to Cochrane Collaboration’s “Risk of bias” tool [13].  Low risk of bias.  High risk of bias.  Unclear risk of bias

The score on the risk of bias [13] achieved by each of the included studies are presented in Fig. 2. The overall quality of RCTs was medium. Eight of the RCTs reported a good randomization procedure [29, 32, 34, 37–40, 42], while the others were ‘unclear’ [23, 25, 26, 30, 31, 33, 35, 36, 41] or ‘high risk’ [24, 27, 28]. Only five studies reported a good allocation concealment [24, 29–31, 40], the other studies were ‘unclear’. Only three studies reported the blinding of the participants [30, 39, 40]. In addition, 12 RCTs reported that the outcome assessors were blinded [26–30, 34, 36–42], while the others were ‘unclear’ [23–25, 31–33, 35, 42]. 15 studies [24–26, 28–31, 34, 35, 37–42] reported the short term withdrawals and the reasons for these dropouts, but only five reported information about the long term withdrawals [26, 28, 37, 38, 40] (all the studies were analyzed on a per protocol basis). Five RCTs did not report a good selective outcome reporting [23, 25, 27, 32, 36].

### Efficacy of AOT

The studies included in this review suggest the efficacy of AOT in improving motor functions both in neurologic and orthopedic diseases. Thirteen articles [23–35] investigated the effectiveness of AOT in post-stroke rehabilitation. Among them, four studies [23–26] showed the efficacy of AOT in improving upper limb functional dexterity in individuals with chronic stroke and two studies in sub-acute subjects [27, 28]. In particular, one study [26] investigated the effects of “standard” AOT (combination of observation and imitation) relative to observation without imitation or execution without observation and to a control group in improving upper limb functional dexterity. This study [26] showed that all the experimental groups (combination, observation and imitation) clinically improved relative to the control group, while no clear difference emerged between the experimental groups. Only one study [29] including acute stroke participants showed a better recovery of functional dexterity in the group performing the conventional therapy. Six articles [30–35] suggest AOT efficacy on walking performance in subjects with chronic stroke and three [31, 33, 35] of them also suggested an efficacy in improving balance. AOT is also recommended in individuals with Parkinson’s disease to improve autonomy in activities of daily living (ADL) [36], to improve spontaneous movement rate of self-paced finger movements [38], and to reduce freezing of gait [37]. Two studies [39, 40] also indicate that AOT improves upper limb motor function in children with cerebral palsy. AOT seems to be effective in improving autonomy in ADL and balance in postsurgical orthopedic subjects [41] (hip fractures or hip or knee replacement) and to enhance knee joint function after total knee replacement [42].

## Discussion

Twenty RCTs were included in this systematic review. The analyzed studies investigated the effects of AOT in improving different motor abilities in diseases like stroke, Parkinson’s disease, cerebral palsy, and postsurgical orthopedic conditions, for a total of 663 subjects. The majority of the studies suggested the efficacy of AOT to improve motor function both in neurologic and orthopedic diseases.

The samples recruited in the most of the RCTs was relatively small and, overall, the quality of the studies was medium. The analysis about the quality highlighted the need to better specify the procedures for allocation concealment, handling of missing data, and blinding of study participants. Lack of uniformity on duration, and frequency of treatments also emerges from included studies, making it difficult to define an optimal posology; the most used duration of a single video is between 3 and 10 min. In keeping with these results and our personal experience, videos lasting 5–6 min seem to be the most reasonable approach to obtain a good balance between individual sustained attention and training efficacy. One month is the most frequent duration of training. In individuals with Parkinson’s disease, the frequency of 3 sessions a week has been suggested to be better than a continuous training because interval times might be necessary for learning consolidation in these subjects [43]. Further studies are needed to determine the optimal frequency, intensity and time of AOT.

Although the studies were too heterogeneous to be pooled, it is interesting to highlight that AOT has an effect in improving motor function regardless of the disease and the severity of motor impairment. Indeed, this approach can be easily adapted to many different conditions, is inexpensive, and can be easily tailored to specific needs of individuals.

No study reported data about the Minimal Clinical Important Difference (MCID) for any outcome measure. We obtained available MCID values from Rehabmeasure.org [44, 45]. Two studies aimed at improving walking ability in chronic stroke individuals achieved mean outcome values equal or greater than the corresponding MCID, i.e., one [33] in the 10MWT (0.l6 m/s) and the other one in the 6 min walking test (89.6 m) and 10MWT (0.36 m/s) [30]. Another study [27], that was designed to investigate the effect of AOT on upper limb functional dexterity in sub-acute stroke participants, achieved a mean Functional Independence Measure score greater than the MCID at two follow-up visits (22.3 and 32.2, respectively).

Probably, the reason why AOT is helpful in addition to a conventional motor training is that it has been shown to facilitate motor learning and the building of a motor memory. It is well known that AOT recruits areas of motor network and MNS such as the ventral premotor cortex, inferior frontal gyrus and IPL, that are activated both during the observation of actions which are part of the motor repertoire of the observer [46] and also in acquiring new motor skills [11]. MNS plays an important part in motor learning [47] that is defined as “a set of processes associated with practice, leading to relatively permanent changes in the capability for movement”. Thus, AOT can be considered as a cognitive tool to improve motor learning [48]. Distinct learning phases can be distinguished in motor learning, from an initial “cognitive” phase, which allows to learn motor sequences, to a retention state in which motor performance can be executed in the absence of any practice after long delay [49]. According to some RCTs showing that motor improvements are maintained after few months, AOT is likely to play a key role in achieving the retention state in comparison to motor training only.

Regarding the type of AOT, it might be interesting to understand the differences between the observation of videos and the observation of an operator performing the action. In fact, the only study [29] showing a better improvement in the control than in the AOT group was characterized by the administration of “observation-to-imitate” training without videos. Moreover, in this study [29], participants observed the operator and performed actions simultaneously. It is unclear whether such a modality of imitation gives the same effects of that after observation. Indeed, it was shown that even splitting the gesture in the simplest movements during the observation facilitates motor learning [49]. Furthermore, the difference with conventional AOT may be that the observation of actions involves a movement imagination processing before the action execution. Motor imagery, together with AOT, can be considered as a “cognitive rehabilitation tool” and plays a key role in motor learning activating MNS regions that are involved in movement preparation and execution [50]. Finally, the maintenance of attention is probably facilitated during the observation of videos.

An open question is about the role played by the different components of AOT: the observation, the imitation and the combined approach. Only one study [26] (see the paragraph ‘Efficacy of AOT’) suggested that action observation without imitation produces effects similar to actual action training, probably through the MN activation, but other studies are needed to deeply investigate the neural substrates underlying these mechanisms.

A potential limitation of our study is the risk of a selection bias because papers for this Review were identified through searches of selected databases (see Search Strategy). In addition, only papers published in English were reviewed.

## Conclusions

In conclusion, data presented in the analyzed articles would suggest that AOT is more beneficial than a simple motor training, enhancing motor recovery regardless of the disease. It could be helpful to design more RCTs combining clinical, imaging and neurophysiological evaluations with the aim to correlate clinical motor changes and cerebral plasticity over time in order to deeply understand the mechanisms underlying motor learning after AOT. Further studies with larger samples, longer follow up and correlations with instrumental data are necessary to define the best way to apply AOT in clinical practice.

## Additional files

Additional file 1: **Features of included studies.** (DOCX 112 kb)

Additional file 2: **Research strategy.** (DOCX 13 kb)
