# Supplementary material for: Action observation training to improve motor function recovery: a systematic review
Source: Arch Physiother. 2015 Dec 2;5:14. doi: 10.1186/s40945-015-0013-x (PMC5759925; doi:10.1186/s40945-015-0013-x)
Supplement: Supplementary file 1 — Features of included studies. (DOCX 112 kb) [file 40945_2015_13_MOESM1_ESM.docx]

**APPENDIX**

**Features of included studies (ordered by pathology)**

**AOT in stroke rehabilitation**

| Harmsen W.J. et al^1^ | |
| --- | --- |
| Aim of the study | To investigate whether a mirror therapy–based action observation (AO) protocol contributes to motor learning of the affected arm after stroke. |
| Participants | 37 participants in the chronic stage after stroke  AOT: n = 18, 9 women, mean ± SD: age 57 ± 10.4  Control: n = 19, 6 women, mean ± SD: age 60 ± 8.8 years  Months from stroke to admission: AOT 46 ± 37, Control 38 ± 25. |
| Intervention | AOT vs control group.  AOT: AOT was administered from a first-person perspective in such a way that the observation of the upper-arm reaching movements was almost similar to what participants would see in the mirror during mirror therapy. To provide participant specific videos, reaching movements from the unaffected arm were videotaped and mirrored. Participants were instructed to focus on the arm reaching movements, which were displayed on the computer monitor. In addition, participants were instructed to observe the AOT with the intention to reproduce the reaching task with the affected arm.  Control: participants in the control group observed a slideshow with static photographs of landscapes as control stimuli. The photographs contained no images of humans or animals and were selected such that they would not trigger the mirror neuron system. |
| Outcome | Outcome measures were assessed pre-intervention and post-intervention.  Because movement time was found to be a predictive measure in learning a simple upper-arm reaching task after stroke it was selected as the primary outcome.  Accelerometer data measured at the arm and target end point were recorded using a TEMEC Vitaport 3 digital recording system. Two 2D acceleration sensors were placed on the ventral side of the wrist, measuring accelerations during the reaching movements in 3 dimensions. |
| Results | Movement time decreased significantly in both groups: 18.3% in the AOT group and 9.1% in the control group. Decrease in movement time was significantly more in the AOT group compared to the control group (p = 0.02). |
| *Risk of bias* | |
| Random sequence generation  (selection bias) | HR – “...To minimize possible confounding effects of motor ability and age, we stratified participants into groups based on age (younger than 55 years of age or 55 years and older) and motor function (FM score <50 or a FM score of ≥50). In this way, we created 4 strata. Stratified randomization was achieved by performing a separate randomization procedure within each stratum. The randomization was based on a computer-generated random code using blocks of 20 patients…” |
| Allocation concealment  (selection bias) | LR – “...The randomization was based on a computer-generated random code using blocks of 20 participants and using sealed envelopes.” |
| Blinding of participants and  personnel (performance bias) | Unclear |
| Blinding of outcome  assessment (detection bias) | Unclear |
| Incomplete outcome data  short term (attrition bias) | LR - No missing outcome data. |
| Incomplete outcome data  long term (attrition bias) | Unclear - The study did not address this outcome. |
| Selective reporting  (reporting bias) | LR - The study protocol is available and all of the study’s pre-specified (primary and secondary) outcomes that are of interest in the review have been reported in the pre-specified way. |
| Other bias |  |

| Park E.C. et al. 2015^2^ | |
| --- | --- |
| Aim of the study | To investigate the effects of AOT on the static balance and walking ability of participants with stroke. |
| Participants | 40 participants diagnosed with hemiplegia resulting from a stroke  AOT: n = 20, 10 women, mean ± SD: age 51.15 ± 14.81, height 163.45 ± 8.53 cm, and weight 64.1 ± 13.1 kg. 11 of the participants had right hemiplegia, and 9 had left hemiplegia.  Control: n = 20, 9 women, mean ± SD: age 48.65 ± 12.81 years, height 165.9 ± 8.6 cm, and weight 63.80 ± 9.26 kg. 9 of the participants had right hemiplegia, and 11 had left hemiplegia.  Months from stroke to admission: AOT 14.8 ± 6.1, Control 13.4 ± 8.2. |
| Intervention | AOT vs control.  Before each training, the participants in both groups received 30 minutes of general physical therapy that consisted of joint mobilization, muscle strengthening and stretching exercises.  AOT: observation of 3-minute videos representing walking on a flat land or on a slope or on steps, all executed by a healthy person; there was one minute of break afterwards. Then, the participants performed walking training for 5 minutes each session, performing the three tasks represented in the videos. Between each set of training, subjects took a 1–2 minute break, and in total, the walking training was about 20 minutes.  Control: participants watched a video showing images of nature unrelated with walking for the same amount of time, and executed the same walking  training performed by the experimental group for 20 minutes.  Session duration: 30 minutes/session, 5 sessions/week, 8 weeks. |
| Outcome | Measures pre-treatment and post-treatment.  Balance ability: analysis system using biofeedback, AP1153 BioRescue, France. The participant was instructed to spread his/her legs to around 30° in an upright standing position and look forward. The participant was instructed to keep his/her balance for one minute while looking forward. The distribution of weight bearing on the paretic and non-paretic side (Limit of stability – LOS), the length (mm), the average speed (Sway Speed – SS in cm/s) , and the area of the movements (Sway Area – SA) were measured.  Gait ability: Timed Up and Go Test (TUG) and 10 Meter Walking Test (10MWT) were assessed. |
| Results | Both groups showed significant improvement in all the measures after the training and the comparison between groups showed significant differences (p < 0.05) in favor of the experimental group for the LOS, SS, SA, TUG and 10MWT but not for the SA. |
| *Risk of bias* | |
| Random sequence generation  (selection bias) | Unclear |
| Allocation concealment  (selection bias) | Unclear |
| Blinding of participants and  personnel (performance bias) | Unclear |
| Blinding of outcome  assessment (detection bias) | Unclear |
| Incomplete outcome data  short term (attrition bias) | Unclear – Insufficient reporting of attrition/exclusion to permit judgement. |
| Incomplete outcome data  long term (attrition bias) | Unclear - The study did not address this outcome. |
| Selective reporting  (reporting bias) | LR - The study protocol is available and all of the study’s pre-specified (primary and secondary) outcomes that are of interest in the review have been reported in the pre-specified way. |
| Other bias |  |

| Kim E. et al 2015^3^ | |
| --- | --- |
| Aim of the study | To investigate the effects of purposeful action observation on upper extremity kinematic patterns in individuals with hemiplegia. |
| Participants | 12 individuals  AOT: n = 6, Control: n = 6 |
| Intervention | AOT group vs control group.  Session duration: 30-minutes daily sessions, 5 sessions/week, 6 weeks  AOT: in the experimental group subjects received traditional occupational treatment and also performed a purposeful action observation training program.  Control: the control group received traditional occupational treatment and topological treatment in which subjects performed purposeful action observation program assignments without actually observing the purposeful actions. |
| Outcome | Measures pre-treatment and post-treatment have been acquired.  A 3-dimensional motion analysis system was used to measure the changes in kinematic patterns of movement of the upper extremity. Three markers were attached to analyze the aiming motion of the upper extremity (average velocity and trajectory ratio). The markers were attached to the side of the index finger proximal interphalangeal and metacarpophalangeal joints, and the wrist. Markers for measuring the range of motion (motion angle) were attached to the dorsal side of the wrist in the middle, the elbow lateral epicondyle, and the insertion of the deltoid muscle. |
| Results | The experimental group showed more improvement than to the control group, but the difference was not statistically significant in the between group comparison (p>0.05). |
| *Risk of bias* | |
| Random sequence generation  (selection bias) | Unclear |
| Allocation concealment  (selection bias) | Unclear |
| Blinding of participants and  personnel (performance bias) | Unclear |
| Blinding of outcome  assessment (detection bias) | Unclear |
| Incomplete outcome data  short term (attrition bias) | LR – No missing outcome data. |
| Incomplete outcome data  long term (attrition bias) | Unclear - The study did not address this outcome. |
| Selective reporting  (reporting bias) | HR - The study protocol is not complete because there are no information about the content of the video-clips. |
| Other bias | The authors don’t provide a table with the comparison of the participants at baseline. |

| Sale et al. 2014^4^ | |
| --- | --- |
| Aim of the study | To discriminate the effectiveness of AOT on upper-limb functional dexterity, in left versus right hemiparetic subjects, in the early phase of stroke onset. |
| Participants | 67 subjects (26 women), Age 66.5 ± 12.7 years  AOT: n = 33, Control: n = 34  Days from stroke to admission: 29.6 ± 4.5  Left hemisphere was involved in 30 cases  All participants were right handed prior to stroke. |
| Intervention | AOT vs control group.  Session duration: two 15-minutes daily sessions, 5 sessions/week, 4 weeks  AOT: observation of video showing 20 different daily routine tasks carried out with the upper limb. 1 task per day. Each action consisted of 3 different motor sequences displayed in order of ascending difficulty and lasting 3 minutes each. The actions were observed from a first-person perspective. At the end of each sequence, the therapist prompted the participant to perform the same movement over 2 minutes, providing help when needed.  Control: participants were shown 5 static images displaying objects (none were animals or people) for 3 minutes. Participants were then asked to perform limb movements as well as feasible for 2 minutes, simulating those performed by the AOT group. |
| Outcome | Measures pre-treatment, post-treatment and at follow up 4 to 5 months after treatment have been acquired.  Fugl-Meyer test for upper limb (FM) and Box and Block test were assessed (BBT was primary outcome to assess the effectiveness of AOT in left versus right hemiparetic subjects). |
| Results | Higher gain for the AOT group than the control group  Action observation, coupled with action execution, induces a higher improvement both in BBT and FM in right hemispheric strokes compared to left hemispheric ones. |
| *Risk of bias* | |
| Random sequence generation  (selection bias) | HR – “…The random allocation to treatment was concealed and based upon a custom computerized system, using dedicated software. Each participating center was provided with client software through which the local participant could ask the server, for any eligible subject, for the group allocation by simply entering his/her age, gender, and brain lesion side…” |
| Allocation concealment  (selection bias) | Unclear |
| Blinding of participants and  personnel (performance bias) | Unclear – The authors speak in general of “…observer-blind trial…” without providing other information. |
| Blinding of outcome  assessment (detection bias) | LR – “…All assessments were performed by a trained occupational therapist (OT) who was not aware of the research aims and treatment content. …” |
| Incomplete outcome data  short term (attrition bias) | LR – No missing outcome data. |
| Incomplete outcome data  long term (attrition bias) | LR – Missing data are balanced in numbers across intervention groups with similar reasons for missing data across groups. |
| Selective reporting  (reporting bias) | LR - The study protocol is available and all of the study’s pre-specified (primary and secondary) outcomes that are of interest in the review have been reported in the pre-specified way. |
| Other bias | The authors don’t provide a table with the comparison of the participants at baseline. |

| Park H.R. et al. 2014^5^ | |
| --- | --- |
| Aim of the study | To assess the efficacy of AOT for walking function of participants with post-stroke hemiparesis. |
| Participants | 21 chronic stroke participants  AOT: n = 11, 3 women, mean age 55.91 ± 9.1  Control: n = 10, 3 women, mean age 54.8 ± 12.22  Months from stroke to admission: AOT 21.09 ± 16.66, Control 25.6 ± 19.67  Capability of independent walking of >10 m with or without walking aids. |
| Intervention | AOT vs control group.  Session duration: 30 minutes/day, 3 times/week for a 4-week period  AOT: observation of 10 minutes of video clips demonstrating four tasks for functional walking (weight shifting to the affected side, walking on straight and curved paths, walking on even and uneven sur­faces, crossing obstacles, and walking with func­tional tasks)  Control: 10 minutes of different land­scape images.  Both groups after observation: 20-min session of walking training. |
| Outcome | Measures pre-treatment and post-treatment  10MWT, Figure-of-8 walk test (F8WT), Dynamic gait index (DGI), and gait symmetry scores. |
| Results | The difference between the pre- and post-test values of the 10MWT, F8WT, and DGI were statistically significantly different between groups (p < 0.05) in favor of the experimental group. |
| *Risk of bias* | |
| Random sequence generation  (selection bias) | LR – “…each subject drew a card from an envelope containing two cards with marking 1 (experimental group) or 2 (control group), without looking at the card.” |
| Allocation concealment  (selection bias) | Unclear – it is unclear whether envelopes were sequentially numbered, opaque and sealed. |
| Blinding of participants and  personnel (performance bias) | HR – this is a single blind RCT. |
| Blinding of outcome  assessment (detection bias) | LR – “The person who performed the measurement and data analysis was blinded to the group of each subject.” |
| Incomplete outcome data  short term (attrition bias) | LR - Missing outcome data balanced in numbers across intervention groups, with similar reasons for missing data across groups. |
| Incomplete outcome data  long term (attrition bias) | Unclear - The study did not address this outcome. |
| Selective reporting  (reporting bias) | LR - The study protocol is available and all of the study’s pre-specified outcomes have been reported in the pre-specified way. |
| Other bias |  |

| Kim J.H. et al. 2013^6^ | |
| --- | --- |
| Aim of the study | To compare the effects of action observation training and motor imagery training on recovery of dynamic balance and gait ability after stroke. |
| Participants | 30 participants after a first-ever stroke over six months since onset  AOT: n = 9, 2 women, mean age 55.3 ± 12.1  MI: n = 9, 3 women, mean age 54.8 ± 8.8  Control: n = 9, 2 women, mean age 59.8 ± 8.9  Onset duration (months): AOT 8.3 ± 3.3, MI 7.3 ± 0.7, Control 8.5 ± 3.6 |
| Intervention | AOT vs MI vs control.  Session duration: 30-minutes daily sessions, 5 sessions/week, 4 weeks  AOT: observation of 20 minutes-video followed by physical training with a therapist for 10 minutes, based on the video. The training program consisted of four stages, one stage for week. Stage 1: pelvic tilting, trunk flexion and extension, and trunk rotation. Stage 2: sit to stand and stand to sit. Stage 3: weight shift. Stage 4: gait.  MI: MI was conducted for 20 minutes through a computer speaker, and the participants then underwent physical training for 10 minutes. The contents of the motor imagery program were identical to the contents suggested in the action observation training program  Control: Physical training  All participants in this study underwent neurodevelopmental therapy for 30 minutes, twice per day, 5 session/week x 4 weeks. The exercise program included training of the trunk, supine to rolling movements, sit to stand, normal gait pattern, training of the lower extremity, weight shifting, and gait level surface and gait stairs. |
| Outcome | Measures pre-treatment and post-treatment: TUG, Functional Reaching Test (FRT), Walking Ability Questionnaire (WAQ), Functional Ambulation Category (FAC) and Spatiotemporal gait parameters. |
| Results | Compared with the physical training group, the AOT group showed significant improvements in the Timed Up and Go test, gait speed, cadence, and single limb support of the affected side. No significant differences in any of the outcome measures were observed between the action observation training group and the motor imagery training group. |
| *Risk of bias* | |
| Random sequence generation  (selection bias) | Unclear |
| Allocation concealment  (selection bias) | LR – “…patients were randomly assigned to select a sealed envelope.” |
| Blinding of participants and  personnel  (performance bias) | Unclear |
| Blinding of outcome  assessment (detection bias) | Unclear |
| Incomplete outcome data  short term (attrition bias) | LR – Missing outcome data balanced in numbers across intervention groups. |
| Incomplete outcome data  long term (attrition bias) | Unclear - The study did not address this outcome. |
| Selective reporting  (reporting bias) | LR - The study protocol is available and all of the study’s pre-specified outcomes have been reported in the pre-specified way. |
| Other bias |  |

| Bang et al. 2013^7^ | |
| --- | --- |
| Aim of the study | To investigate the effect of AOT on walking ability with chronic stroke participants. |
| Participants | 30 chronic stroke participants  AOT: n = 15, 6 women, mean age 64.1 ± 6.35  Control: n = 15, 7 women, mean age 58.9 ± 6.03  Time after stroke (months): AOT 14.1 ± 3.78, Control 12.6 ± 3.86  Gait speed over 0.5 m/s, enough to exercise on the treadmill. |
| Intervention | AOT vs control group.  Session duration: 40 minutes/day, 5 times/week for a 4-week period.  AOT: treadmill training (30 min) after watching the treadmill video (9 min).  Control: treadmill training (30 min) after watching the nature video (9 min). |
| Outcome | Measures pre-treatment and post-treatment were acquired.  TUG, 10MWT, 6-minute walk test (6MWT), Knee angle in swing phase during walking. |
| Results | Both groups showed a significant improvement after treatment. All the outcome measures showed a significant difference between groups (p < 0.05) in favor of the experimental group. |
| *Risk of bias* | |
| Random sequence generation  (selection bias) | Unclear |
| Allocation concealment  (selection bias) | LR – “...selection from opaque closed envelopes containing the group assignment”. |
| Blinding of participants and  personnel (performance bias) | LR – “This study was a double-blind randomized controlled trial. Both the assessor and the participants were blinded.” |
| Blinding of outcome  assessment (detection bias) | LR – “This study was a double-blind randomized controlled trial. Both the assessor and the participants were blinded. The assessor was not involved in the study”. |
| Incomplete outcome data  short term (attrition bias) | LR – “All participants completed the entire study”. |
| Incomplete outcome data  long term (attrition bias) | Unclear – The study did not address this outcome. |
| Selective reporting  (reporting bias) | LR - The study protocol is available and all of the study’s pre-specified outcomes have been reported in the pre-specified way. |
| Other bias |  |

| Park C.S. et al. 2013^8^ | |
| --- | --- |
| Aim of the study | To evaluate the functional effects of an additional action observational training to a functional electrical stimulation treatment on weight bearing, stability and gait velocity of hemiplegic participants. |
| Participants | 20 subjects more than six months post-stroke participated.  AOT: n = 10, 4 female  Control: n = 10, 5 female |
| Intervention | Both groups received functional electrical stimulation treatment. The experimental group additionally received AOT. They were asked to watch a video on gait for 15 minutes. The video on gait showed walking on flat ground, slopes, and stairs, and was watched 5 times a week for 6 weeks. |
| Outcome | Balance and gait velocity were measured at the baseline, and after six weeks of treatment. |
| Results | The experimental group showed significant increases in weight bearing (anterior-posterior, right-left) on the affected side, stability index and gait velocity. Moreover, according to the comparison of training effects between in the two groups, the variables of anterior-posterior weight bearing, stability index and gait velocity revealed a statistically significant difference. |
| *Risk of bias* | |
| Random sequence generation  (selection bias) | Unclear |
| Allocation concealment  (selection bias) | Unclear |
| Blinding of participants and  personnel  (performance bias) | Unclear |
| Blinding of outcome  assessment (detection bias) | Unclear |
| Incomplete outcome data  short term (attrition bias) | LR – no missing outcome data. |
| Incomplete outcome data  long term (attrition bias) | Unclear – The study did not address this outcome. |
| Selective reporting  (reporting bias) | LR - The study protocol is available and all of the study’s pre-specified outcomes have been reported in the pre-specified way. |
| Other bias |  |

| Lee D. et al. 2013^9^ | |
| --- | --- |
| Aim of the study | This study examined the effects of action observation and action practice on stroke participants’ upper limb function. |
| Participants | 33 chronic stroke participants  Action observation group: n = 8 (3 F), mean age: 63±3.7 years  Action practice group: n = 9 (4 F), mean age: 62±1.5 years  Action observation + action practice group: n = 9 (4 F), mean age: 61±2.3 years  Control group: n = 7 (4 F), mean age: 60±5.9 years. |
| Intervention | The action observation group watched a video of the task, the action practice group performed the action, the combined action observation-action practice group watched the video of the task and practiced the action, and the control group did not perform either action observation or action practice. The video used in the action observational physical training comprised a scene of an adult male picking up a cup, bringing it to his mouth in order to touch his mouth, and then returning the cup to its initial position. The action observation group watched the video of the task on a large screen for 10 minutes in a quiet room. The action practice group performed the action of picking up an empty cup for 10 minutes. The combined action observation–action practice group observed the task video for five minutes and practiced the action for five minutes. |
| Outcome | The assessment was performed three times: before the intervention, immediately after the completion of the intervention, and one week later. In the assessment, the number of times the full drinking action was performed in one minute was measured. Only complete actions within the measuring time were counted, and those that were not completed were excluded from the measurement. |
| Results | Improvements in drinking behavior functions were observed immediately after the experiment and one week later. After the intervention, the number of drinking motions had increased the most in the combination group. A combination of action observation and action training is the most effective treatment method, and action training is a desirable second to combined therapy. |
| Risk of bias | |
| Random sequence generation  (selection bias) | Unclear |
| Allocation concealment  (selection bias) | Unclear |
| Blinding of participants and  personnel  (performance bias) | Unclear |
| Blinding of outcome  assessment (detection bias) | LR – “The physical therapist who conducted the assessments did not have any information about the experimental group of the subjects”. |
| Incomplete outcome data  short term (attrition bias) | LR – no missing outcome data. |
| Incomplete outcome data  long term (attrition bias) | LR – no missing outcome data. |
| Selective reporting  (reporting bias) | LR - The study protocol is available and all of the study’s pre-specified outcomes have been reported in the pre-specified way. |
| Other bias |  |

| Cowles et al. 2013^10^ | |
| --- | --- |
| Aim of the study | To investigate whether Observation-to-Imitate (OTI) followed by physical practice (OTI + PP) enhanced the benefits of conventional physical therapy (CPT) on upper limb recovery early after stroke. |
| Participants | 29 participants who had suffered a stroke between 3 and 31 days before recruitment OTI + PP: n = 15, 7 women, mean age 78.8 ± 8.1  CPT: n = 14, 5 women, mean age 75.6 ± 12.4  Days from stroke to admission: OTI + PP 19.5 ± 7.0, Control 17.8 ± 5.1. |
| Intervention | OTI + PP vs control.  AOT: OTI + PP was provided in two 30-minute sessions, separated by a 10-minutes rest session, every day for 15 working days. The activities showed in the videos were functional activities that people need to relearn after stroke: bring telephone to ear, cleaning a table… The instruction was to watch the research therapist performing the functional task for 1 to 2 minutes and to think about copying in preparation for doing exactly the same in time with the therapist for 4 to 6 minutes. Verbal correction was given but no physical contact was made.  Control: no therapy in addition to CPT. |
| Outcome | Measures pre-treatment and post-treatment were acquired.  Primary outcome: Motricity index (MI) to assess the ability to voluntarily contract paretic muscle.  Secondary outcome: Action Research Arm Test (ARAT) to assess the functional use of upper limb. |
| Results | Improvements over time for FAT, FM, FIMM and BBT in both groups have been found.  Significant Time × Treatment interaction at Box and Block test in AOT group has been shown, resulting in a positive effect of AOT on the functional dexterity in acute stroke participants. |
| *Risk of bias* | |
| Random sequence generation  (selection bias) | LR – “Group allocation was computer generated in blocks of 4 by a university statistician before the trial began”. |
| Allocation concealment  (selection bias) | LR- “Details of group allocation were placed into sequentially numbered, opaque, sealed envelopes. These were held by an independent administrator. After a participant had completed baseline measures, the research therapist telephoned the administrator who then opened the relevant envelope and divulged participant allocation details”. |
| Blinding of participants and  personnel  (performance bias) | Unclear – The authors speak in general of “…observer-blind trial…” without providing other information. |
| Blinding of outcome  assessment (detection bias) | LR – “All measures were made by an assessor blinded to treatment allocation”. |
| Incomplete outcome data  short term (attrition bias) | HR – Imbalance in number for missing data across intervention groups. |
| Incomplete outcome data  long term (attrition bias) | Unclear – The study did not address this outcome. |
| Selective reporting  (reporting bias) | LR - The study protocol is available and all of the study’s pre-specified outcomes have been reported in the pre-specified way. |
| Other bias |  |

| Franceschini et al. 2012^11^ | |
| --- | --- |
| Aim of the study | To assess the efficacy of AOT on the recovery of upper-limb functional dexterity after stroke. |
| Participants | 102 stroke survivors from 13 centers 30 days (± 7) after a first-ever stroke  AOT: n = 53, 20 women, mean age 67.0 ± 12.4,  Control: n = 49, 21 women, mean age 65.7 ± 11.9  Days from stroke to admission: AOT 31.0 ± 4.6, Control 29.5 ± 4.2  All participants were right handed prior to stroke. |
| Intervention | AOT vs control.  Session duration: two 15-minutes daily sessions, 5 sessions/week, 4 weeks  AOT: observation of video showing 20 different daily routine tasks carried out with the upper limb. 1 task per day. Each action consisted of 3 different motor sequences displayed in order of ascending difficulty and lasting 3 minutes each. The actions were observed from a first-person perspective. At the end of each sequence, the therapist prompted the participant to perform the same movement over 2 minutes, providing help when needed.  Control: participants were shown 5 static images displaying objects (none were animals or people) for 3 minutes. Participants were then asked to perform limb movements as well as feasible for 2 minutes, simulating those performed by the AOT group. |
| Outcome | Measures pre-treatment, post-treatment and at follow up 4 to 5 months after treatment were acquired.  Fugl-Meyer test (FM), Frenchay Arm test (FAT), Box and Block test (BBT), Modified Ashworth Scale (MAS), and Functional Independence Measure – Motor score (FIMM). |
| Results | Improvements over time for FAT, FM, FIMM and BBT in both groups  Significant Time × Treatment interaction at Box and Block test in AOT Group  Positive effect of AOT on the functional dexterity of sub-acute stroke participants. |
| *Risk of bias* | |
| Random sequence generation  (selection bias) | HR – “…The random allocation to treatment was concealed and based on a custom computerized system. Each participating center asked the server for the group allocation after typing in age, gender, and brain lesion side…”. |
| Allocation concealment  (selection bias) | Unclear |
| Blinding of participants and  personnel  (performance bias) | Unclear |
| Blinding of outcome  assessment (detection bias) | LR – “…All assessments were performed by trained professionals not involved in the research treatment and blind to group allocation…”. |
| Incomplete outcome data  short term (attrition bias) | Unclear – No reasons for missing data provided. |
| Incomplete outcome data  long term (attrition bias) | Unclear – No reasons for missing data provided. |
| Selective reporting  (reporting bias) | HR – The study doesn’t report the intention-to-treat analysis. “…The results (not shown) agreed with the models fitted to the dataset of observed data…”. The authors report Box and Block test as primary outcome without providing a clear justification. |
| Other bias |  |

| Kim J.S. et al. 2012^12^ | |
| --- | --- |
| Aim of the study | To investigate the effects of AOT on walking performance of stroke participants with hemiparesis. |
| Participants | 30 participants  AOT: n = 15, mean age 64.1 ± 8.3  Control: n = 15, mean age 65.5 ± 7.7  Months from stroke to admission: AOT 4.6 ± 8.3, Control 4.1 ± 1.0. |
| Intervention | AOT vs control.  30 minutes of physical therapy training in both groups.  AOT: observation of 5 videos lasting each one 2 minutes and representing anterior, side and posterior view of walking straight with long steps; all direction view of walking straight with long steps with in slow and fast motion. After video-clips participants had to walk for 10 minutes.  Control: 10 minutes of video in which they were taken through a progressive relaxation program (stretching) after each therapy session. |
| Outcome | Measures pre-treatment and post-treatment were acquired.  Spatiotemporal gait parameters measured by the GAITRite system: affected side step length and stride length, affected side single support time, double support time, gait velocity and cadence. |
| Results | Only the AOT group showed significant improvement in all these measures after the training and the comparison with the control group after training showed significant differences between groups |
| *Risk of bias* | |
| Random sequence generation  (selection bias) | LR – “…patients were randomly assigned to the experimental or control groups by a computerized number-generator performed by an independent researcher.” |
| Allocation concealment  (selection bias) | Unclear |
| Blinding of participants and  personnel (performance bias) | Unclear |
| Blinding of outcome  assessment (detection bias) | Unclear |
| Incomplete outcome data  short term (attrition bias) | Unclear |
| Incomplete outcome data  long term (attrition bias) | Unclear - The study did not address this outcome. |
| Selective reporting  (reporting bias) | HR - The study protocol is not complete because there are no information about the frequency and the lasting of the treatment. The content of the video-clip watched by the control group is not described. |
| Other bias | The motor training after video-clip session is not the same in the two groups (walking training in the AOT group VS stretching training in the control group). |

| Ertelt et al. 2007^13^ | |
| --- | --- |
| Aim of the study | To assess whether action observation therapy may lead to clinical improvement of motor impairment of upper limb in chronic stroke participants. |
| Participants | 15 participants (4 women) with a confirmed diagnosis of a first ever ischemic stroke sustained more than 6 months before entering the study  AOT: n = 7, 2 women, mean age 57.16 ± 8.73  Control: n = 8, 2 women, mean age 55.40 ± 10.77  Days from stroke to admission: AOT 1472.9 ± 1258.8, Control 724.8 ± 360.9  All participants had a moderate paresis of the contralateral upper limb as assessed by standard functional scales. |
| Intervention | AOT vs control group.  Session duration: 18 rehabilitation sessions of 90 minutes on 18 consecutive working days.  AOT: careful observation on a large TV screen of video sequences containing daily life hand and arm actions. After the observation of the video sequence for 6 minutes, the participant was asked to perform the observed action at the best convenience for 6 minutes with his/her paretic upper limb using the same objects as shown in the video. Every day a “unit” of three hand and/or arm movements of increasing complexity were presented, for a total of fifty-four different video sequences presented.  Control: participants entering the control group matched the experimental treatment with the exception that the participants watched sequences of geometric symbols and letters instead of action sequences. |
| Outcome | Measures baseline (14 days before the onset of the AOT), pre-treatment, post-treatment and at follow up 8 weeks after the end of the intervention (only for AOT group) were acquired.  Standard functional scales: Wolf Motor Function Test (WMFT) and FAT Subjective scale: Stroke Impact Scale (SIS) . |
| Results | Comparison of the test scores before and after the treatment: significant differences in the AOT group (p < 0.05) but not in the control group.  Analysis of the differences between post-test and pre-test measurements between the experimental and the control group: significant difference for the FAT and the SIS (p < 0.01) the but not for the WMFT. |
| *Risk of bias* | |
| Random sequence generation  (selection bias) | Unclear |
| Allocation concealment  (selection bias) | Unclear |
| Blinding of participants and  personnel  (performance bias) | Unclear |
| Blinding of outcome  assessment (detection bias) | Unclear – Insufficient information provided. |
| Incomplete outcome data  short term (attrition bias) | Unclear – No reasons for missing data provided. |
| Incomplete outcome data  long term (attrition bias) | Unclear - No reasons for missing data provided. |
| Selective reporting  (reporting bias) | HR – The authors report a significant difference of the WMFT comparison that is in conflict with the result reported in the table. |
| Other bias | It is not clear how the authors demonstrate that the neurological impairment of participants is stable. They report p < 0.4 instead of p > 0.05. |

**AOT in Parkinson’s disease**

| Pelosin E. et al. 2013^14^ | |
| --- | --- |
| Aim of the study | To understand the effects of action observation on the spontaneous rate of finger movements in participants with PD. |
| Participants | 38 individuals (21 women) with PD. Age: 67.4 ± 7.4  14 matched healthy controls (7 women). |
| Intervention | Participants in the VIDEO group watched video clips showing repetitive finger movements paced at 3 Hz, whereas those in the ACOUSTIC group listened to an acoustic cue paced at 3 Hz. All participants performed a finger sequence at their spontaneous pace at different intervals (before, at the end of, 45 minutes after, and 2 days after training); 8 participants with PD were recruited for a sham intervention, watching a 6-minute video representing a static hand. Finally, 10 participants participated in the same protocol used for the VIDEO group but were tested in the on and off medication states. |
| Outcome | The main outcome measure in all the experiments was the spontaneous movement rate (SMR) of self-paced finger movements. The motor task consisted in the execution of 2 repetitions of the same sequence of finger opposition movements (opposition of thumb to index, medium, ring, and little fingers) lasting 1 minute, separated by a 1-minute rest. Participants wore a sensor-engineered glove on their right hand (Glove analyzer System [GAS], eTT s.r.l., Italy). Data were acquired at 1 kHz (National Instrument board 800008B-01). |
| Results | Both video and acoustic training increased the SMR, but training with video was more effective than acoustic training in modifying the motor performance at longer intervals (45 minutes and 2 days after training). Post hoc analysis revealed that video observation induced a decrease of intertapping interval larger than acoustic modality at all the testing times.  Control experiment 1 showed that the mere observation of a static image did not induce any modifications in motor performance in PD participants.  Control experiment 2 showed that the SMR increased after action observation training in both conditions (ON and OFF) but no significant difference was found at POST. |
| Risk of bias | |
| Random sequence generation  (selection bias) | LR – “The randomization sequence was created using computer-generated random number tables with 1:1:1:1 allocation of PD patients and 1:1 allocation of NCs. An independent researcher did the randomization and advised the therapists about group allocation…”. |
| Allocation concealment  (selection bias) | Unclear – No sufficient information provided. |
| Blinding of participants and  personnel  (performance bias) | Unclear |
| Blinding of outcome  assessment (detection bias) | LR – “The referring neurologist and the researcher conducting the assessments were kept blind to group allocation”. |
| Incomplete outcome data  short term (attrition bias) | LR – no missing outcome data. |
| Incomplete outcome data  long term (attrition bias) | LR – no missing outcome data. |
| Selective reporting  (reporting bias) | LR - The study protocol is available and all of the study’s pre-specified outcomes have been reported in the pre-specified way. |
| Other bias |  |

| Buccino et al. 2011^15^ | |
| --- | --- |
| Aim of the study | To assess the benefits of AOT, in addition to conventional rehabilitation, on the autonomy in ADL in PD participants. |
| Participants | 15 PD participants  AOT: n = 7, 2 women, mean age 68 (59-80)  Control: n = 8, 3 women, mean age 73.5 (67.5–76.5)  Occurrence of freezing at least once a week and for at least 2 sec: minimum score of 2 and 1 on item 3 and 4 respectively at the FOG Questionnaire; FOG-Q Mini mental state examination (MMSE) > 24. |
| Intervention | AOT vs control group.  AOT: observation and subsequently execution of different daily actions presented through video clips.  Control: observation of video clips with no motor content and subsequently performance of the same actions as cases. |
| Outcome | Measures pre-treatment and post-treatment were acquired.  Autonomy in ADL: UPDRS and FIM. |
| Results | Both groups improved after treatment.  Functional score gain was higher in cases than in controls on both the UPDRS (P = 0.004) and the FIM (P = 0.002).  AOT may improve autonomy in daily activities in PD participants. |
| *Risk of bias* | |
| Random sequence generation  (selection bias) | Unclear |
| Allocation concealment  (selection bias) | Unclear |
| Blinding of participants and  personnel (performance bias) | Unclear - Insufficient information provided. |
| Blinding of outcome  assessment (detection bias) | LR – “Functional evaluation was carried out by a physician blinded to group assignment…”. |
| Incomplete outcome data  short term (attrition bias) | Unclear |
| Incomplete outcome data  long term (attrition bias) | Unclear - The study did not address this outcome. |
| Selective reporting  (reporting bias) | HR - The study protocol is not complete because there are no information about the frequency and the lasting of the treatment. |
| Other bias |  |

| Pelosin et al. 2010^16^ | |
| --- | --- |
| Aim of the study | To assesses the efficacy of AOT in reducing freezing of gait (FoG) in participants with Parkinson’s Disease. |
| Participants | 18 participants (8 women) with Parkinson’s Disease (PD)  AOT: n = 9, mean age 68.8 ± 4.1  Control: n = 9, mean age 70.2 ± 6.8  Occurrence of freezing at least once a week and for at least 2 sec: minimum  score of 2 and 1 on item 3 and 4 respectively at the FOG Questionnaire;  Mini mental state examination (MMSE) > 24. |
| Intervention | AOT vs control.  Session duration: 60-minute training for 3 sessions a week for 4 weeks  Execution of the same motor training and in addiction  AOT: observation of video-clips representing the same actions that they performed. Each clip lasting 6 minutes and in a training session 2 video-clips were presented twice. After the video observation participants were asked to practice (for a total amount of 36 minutes of execution)  Control: Observation of landscape videos and execution of same exercises in the exact order and for the same amount of time than AOT group. |
| Outcome | Measures pre-treatment, post-treatment and at follow-up 4 weeks after the end of training (FU-4).  FoG-Q and FoG-diary to evaluate FoG frequency and severity.  H&Y to evaluate the disease severity.  UPDRSIII to evaluate the motor impairment.  TUG and 10MWT to evaluate the walking performance.  Berg Balance Scale and Tinetti score to evaluate the static and dynamic performance.  PDQ-39 to assess the quality of life. |
| Results | Significant reduction of FOG Questionnaire score and number of FOG episodes in both groups. At FU-w4 examination, a significant reduction in the number of FOG episodes was observed only in the Action group. Motor performance (walking and balance) and quality-of-life assessments were significantly improved in both groups at the end of training and at follow-up. |
| *Risk of bias* | |
| Random sequence generation  (selection bias) | LR – “…patients were randomly assigned to the experimental (labeled “Action”) or control (labelled “Landscape”) groups by a computerized random-number generator performed by an independent researcher.” |
| Allocation concealment  (selection bias) | Unclear |
| Blinding of participants and  personnel (performance bias) | HR – this is a single bind RCT |
| Blinding of outcome  assessment (detection bias) | LR – “Patients’ evaluation was performed by a blinded rater, unaware of group allocation.” |
| Incomplete outcome data  short term (attrition bias) | LR – No missing outcome data |
| Incomplete outcome data  long term (attrition bias) | LR – No missing outcome data |
| Selective reporting  (reporting bias) | LR - The study protocol is available and all of the study’s pre-specified outcomes have been reported in the pre-specified way. |
| Other bias |  |

**AOT in children with cerebral palsy**

| Sgandurra et al. 2013^17^ | |
| --- | --- |
| Aim of the study | To assess the effects of AOT on upper limb function in children with unilateral CP. |
| Participants | 24 children (8 females) between 5 and 15 years, grade between 4 and 8 on the House Functional Classification System (HFCS)  AOT: n = 12, 4 females, mean age 9.48 ± 2.12, right hemiplegia n =10,  HCFS: 5.58 ± 1.31  Control: n = 12, 4 females, mean age 9.94 ± 2.77, right hemiplegia n =7  HCFS: 5.75 ± 1.36 |
| Intervention | AOT vs control group.  Session duration: 15 rehabilitation sessions (each session lasting 60 minutes) on 15 consecutive working days for a total of 45 different goal-directed actions. 3 actions for each session.  AOT: observation (3 minutes) of video sequences showing unimanual or bimanual goal directed actions (like fill up a glass of water or pick up a coin and put it in a money box) followed by the execution of the observed actions (3 minutes).  Control: observation of computer games and then performance of the same upper limb actions and in the same order as the experimental group. |
| Outcome | Measures before the training (T0), and at 1 week (T1), 8 weeks (T2), and 24 weeks (T3) after the end of the training.  Primary outcome measure: Assisting Hand Assessment (AHA) test.  Secondary outcome: Melbourne Assessment of Unilateral Upper Limb Function (MUUL).  Parents filled out the ABILHAND-Kids questionnaire. |
| Results | The experimental group improved more in score changes for the AHA at the primary endpoints T1 (P = .008), T2 (P = .019), and T3 (P = .049).  No between-group significant changes were found for ABILHAND-Kids or Melbourne assessment. |
| *Risk of bias* | |
| Random sequence generation  (selection bias) | LR – “…children will be block randomized into pairs…using a computer generated set of random numbers. A computer random allocation will also be used to randomly assign the subjects of each pair to experimental or control group by a computer random allocation.” |
| Allocation concealment  (selection bias) | LR – “The master list of random numbers will be located in locked cabinets only accessible at completion of the RCT for analysis.” |
| Blinding of participants and  personnel (performance bias) | LR – In order to make blind the study the children enrolled and their parents will be informed about general description of the design of the study…but not the kind of observation…The treating therapists and study personnel, committed to help during the treatment, will be not blinded of group allocation.” |
| Blinding of outcome  assessment (detection bias) | LR – “Outcome measures will be administered by a therapist blind of group assignment and scored by different assessors also blind to group allocation.” |
| Incomplete outcome data  short term (attrition bias) | LR – No missing data. |
| Incomplete outcome data  long term (attrition bias) | LR – No missing data. |
| Selective reporting  (reporting bias) | LR - The study protocol is available and all of the study’s pre-specified outcomes have been reported in the pre-specified way. |
| Other bias |  |

| Buccino et al. 2012^18^ | |
| --- | --- |
| Aim of the study | To assess whether AOT may improve upper limb motor functions in children with cerebral palsy (CP). |
| Participants | 15 children (6 females), between 6 and 11 years, IQ > 70  AOT: n = 8, 4 women  Control: n = 7, 2 women  Six participants had left-sided hemiplegia, six right-sided hemiplegia, three had tetraplegia. |
| Intervention | AOT vs control group.  Session duration: 5 times a week for 3 consecutive weeks.  AOT: daily observation of one clip (9-12 minutes) showing a specific daily action requiring the use of the arms and /or hands (using a pencil, playing with Lego). Each action was subdivided into three or four constituent motor segments and presented from different perspectives (3 minutes). Subsequently performance of the same actions (2 minutes for each action).  Control: observation of video clips addressing various topics (geography, history, science adapted for children), but with no motor content. Execution of the same actions as children in the case group for the same duration. |
| Outcome | Measures twice (T1, T2) at baseline (2weeks apart) and at the end of the treatment (T3).  Primary outcome measure: Melbourne Assessment Scale (MaS). |
| Results | Baseline: groups did not differ on functional evaluation  Post-treatment: the functional score gain was significantly better in the case group than control group (p=0.026). |
| *Risk of bias* | |
| Random sequence generation  (selection bias) | LR – “Enrolled children were randomly allocated to the case …or the control group…by means of dedicated software.” |
| Allocation concealment  (selection bias) | Unclear |
| Blinding of participants and  personnel (performance bias) | LR – “The children and their parents were blind to group allocation.” |
| Blinding of outcome  assessment (detection bias) | LR – “…functional assessment at times T1, T2, and T3…was carried out by a physician blind to treatment allocation…”. |
| Incomplete outcome data  short term (attrition bias) | LR – No missing data |
| Incomplete outcome data  long term (attrition bias) | Unclear - The study did not address this outcome. |
| Selective reporting  (reporting bias) | LR - The study protocol is available and all of the study’s pre-specified outcomes have been reported in the pre-specified way. |
| Other bias |  |

**AOT in orthopedic patients**

| Park S.D. et al. 2014^19^ | |
| --- | --- |
| Aim of the study | To investigate the effects of AOT on knee joint function and balance in total knee replacement (TKR) participants. |
| Participants | 18 participants who were diagnosed with degenerative gonarthritis and underwent TKR.  AOT: n = 9, mean age 72.67 ± 12.25  Control: n = 9, mean age 70.56 ± 10.98. |
| Intervention | AOT vs control group.  Session duration: once per day, three times per week, for three weeks All participants underwent conventional physical therapy (30 minutes of gait exercise and tread­mill).  AOT: observation (10 minutes) of video clips showing daily actions (e.g. raising legs in a supine posi­tion, sitting and standing without flexing the waist) and subsequently execution (30 minutes) of the same actions.  Control: execution of the same actions as participants in the AOT group (30 minutes). |
| Outcome | Measures pre-treatment and post-treatment were acquired.  Western Ontario and Mc-Master Universities Osteoarthritis Index (WOMAC), including pain (VAS), stiffness and function; TUG. |
| Results | After intervention both participants in the AOT and in the control group improved significantly in all outcome measures in a within-group comparison. The between-group comparisons showed no significant difference for the TUG test while for the WOMAC there were significant differences in favor of the AOT group. |
| *Risk of bias* | |
| Random sequence generation  (selection bias) | LR – “The subjects were ran­domly allocated to the action observation training group and the PT group through coin flipping.” |
| Allocation concealment  (selection bias) | Unclear |
| Blinding of participants and  personnel (performance bias) | Unclear |
| Blinding of outcome  assessment (detection bias) | Unclear |
| Incomplete outcome data  short term (attrition bias) | LR – No missing data. |
| Incomplete outcome data  long term (attrition bias) | Unclear - The study did not address this outcome. |
| Selective reporting  (reporting bias) | LR - The study protocol is available and all of the study’s pre-specified outcomes have been reported in the pre-specified way. |
| Other bias |  |

| Bellelli et al. 2010^20^ | |
| --- | --- |
| Aim of the study | To assess whether AOT may also improve motor recovery in postsurgical orthopedic participants in addition to conventional physiotherapy. |
| Participants | 60 participants (age = 18-90 years) admitted to a post-orthopedic (hip fractures or hip or knee replacement) surgery rehabilitation  AOT: n = 30, 21 women, mean age 71.9 ± 8.4, hip arthroplasty n = 15, knee arthroplasty n = 12, hip fracture repair n = 3  Control: n = 30, 16 women, mean age 71.8 ± 6.9, hip arthroplasty n = 10, knee arthroplasty n = 18, hip fracture repair n = 2. |
| Intervention | AOT vs control group.  Both groups participated in a conventional post-orthopedic rehabilitation program for 1 hour a day, 6 days a week, for 3 weeks.  AOT: additional daily session of 24 minutes during which they were asked to observe 3 short movies showing an actor performing daily actions with the leg or trunk; each short movie included 4 different 2-minute actions. After observation, participants were required to execute the observed actions to the best of their ability.  Control: observation of movies showing scenes with no motor content (geographic documentaries) and execution of the same actions as participants in the case group. |
| Outcome | Measures at baseline, and at 1 week (T1), 2 weeks (T2), and 3 weeks (post-treatment, T3).  FIM scores, FIM motor sub-scores, Tinetti scores and types of walking aids for gait from admission to discharge. |
| Results | Changes in FIM scores and Tinetti absolute functional gain, FIM absolute functional efficiency, and FIM motor sub-scores were significantly greater in participants of the case groups than in participants of the control groups.  At discharge, participants of the AOT group were prescribed a single crutch for all except 1 person. This measure of change was significantly different between groups (8 participants of the control group were prescribed 2 crutches at discharge, p = 0.01). |
| *Risk of bias* | |
| Random sequence generation  (selection bias) | Unclear |
| Allocation concealment  (selection bias) | Unclear |
| Blinding of participants and  personnel (performance bias) | Unclear |
| Blinding of outcome  assessment (detection bias) | LR – “...functional status was assessed by a physician who was blinded to treatment allocation.” |
| Incomplete outcome data  short term (attrition bias) | LR – No missing data |
| Incomplete outcome data  long term (attrition bias) | Unclear - The study did not address this outcome. |
| Selective reporting  (reporting bias) | LR - The study protocol is available and all of the study’s pre-specified outcomes have been reported in the pre-specified way. |
| Other bias |  |

**References**

1. Harmsen WJ, Bussmann JB, Selles RW, Hurkmans HL, Ribbers GM. A Mirror Therapy-Based Action Observation Protocol to Improve Motor Learning After Stroke. Neurorehabil Neural Repair 2015;29:509-516.

2. Park EC, Hwangbo G. The effects of action observation gait training on the static balance and walking ability of stroke patients. J Phys Ther Sci 2015;27:341-344.

3. Kim E, Kim K. Effects of purposeful action observation on kinematic patterns of upper extremity in individuals with hemiplegia. J Phys Ther Sci 2015;27:1809-1811.

4. Sale P, Ceravolo MG, Franceschini M. Action Observation Therapy in the Subacute Phase Promotes Dexterity Recovery in Right-Hemisphere Stroke Patients. Biomed Research International 2014.

5. Park HR, Kim JM, Lee MK, Oh DW. Clinical feasibility of action observation training for walking function of patients with post-stroke hemiparesis: a randomized controlled trial. Clin Rehabil 2014;28:794-803.

6. Kim JH, Lee BH. Action observation training for functional activities after stroke: a pilot randomized controlled trial. NeuroRehabilitation 2013;33:565-574.

7. Bang DH, Shin WS, Kim SY, Choi JD. The effects of action observational training on walking ability in chronic stroke patients: a double-blind randomized controlled trial. Clin Rehabil 2013;27:1118-1125.

8. Park CS, Kang KY. The effects of additional action observational training for functional electrical stimulation treatment on weight bearing, stability and gait velocity of hemiplegic patients. J Phys Ther Sci 2013;25:1173-1175.

9. Lee D, Roh H, Park J, Lee S, Han S. Drinking behavior training for stroke patients using action observation and practice of upper limb function. J Phys Ther Sci 2013;25:611-614.

10. Cowles T, Clark A, Mares K, Peryer G, Stuck R, Pomeroy V. Observation-to-Imitate Plus Practice Could Add Little to Physical Therapy Benefits Within 31 Days of Stroke: Translational Randomized Controlled Trial. Neurorehabil Neural Repair 2013;27:173-182.

11. Franceschini M, Ceravolo MG, Agosti M, et al. Clinical Relevance of Action Observation in Upper-Limb Stroke Rehabilitation: A Possible Role in Recovery of Functional Dexterity. A Randomized Clinical Trial. Neurorehabil Neural Repair 2012;26:456-462.

12. Kim JS, Kim K. Clinical Feasibility of Action Observation Based on Mirror Neuron System on Walking Performance in Post Stroke Patients. J Phys Ther Sci 2012;24:597-599.

13. Ertelt D, Small S, Solodkin A, et al. Action observation has a positive impact on rehabilitation of motor deficits after stroke. Neuroimage 2007;36:T164-T173.

14. Pelosin E, Bove M, Ruggeri P, Avanzino L, Abbruzzese G. Reduction of Bradykinesia of Finger Movements by a Single Session of Action Observation in Parkinson Disease. Neurorehabil Neural Repair 2013;27:552-560.

15. Buccino G, Vogt S, Ritzl A, et al. Neural circuits underlying imitation learning of hand actions: an event-related fMRI study. Neuron 2004;42:323-334.

16. Pelosin E, Avanzino L, Bove M, Stramesi P, Nieuwboer A, Abbruzzese G. Action observation improves freezing of gait in patients with Parkinson's disease. Neurorehabil Neural Repair 2010;24:746-752.

17. Sgandurra G, Ferrari A, Cossu G, Guzzetta A, Fogassi L, Cioni G. Randomized trial of observation and execution of upper extremity actions versus action alone in children with unilateral cerebral palsy. Neurorehabil Neural Repair 2013;27:808-815.

18. Buccino G, Arisi D, Gough P, et al. Improving upper limb motor functions through action observation treatment: a pilot study in children with cerebral palsy. Dev Med Child Neurol 2012;54:822-828.

19. Park SD, Song HS, Kim JY. The effect of action observation training on knee joint function and gait ability in total knee replacement patients. J Exerc Rehabil 2014;10:168-171.

20. Bellelli G, Buccino G, Bernardini B, Padovani A, Trabucchi M. Action observation treatment improves recovery of postsurgical orthopedic patients: evidence for a top-down effect? Arch Phys Med Rehabil 2010;91:1489-1494.
