# Supplementary material for: Action observation training to improve motor function recovery: a systematic review
Source: Arch Physiother. 2015 Dec 2;5:14. doi: 10.1186/s40945-015-0013-x (PMC5759925; doi:10.1186/s40945-015-0013-x)
Supplement: Supplementary file 2 — Research strategy. (DOCX 13 kb) [file 40945_2015_13_MOESM2_ESM.docx]

(Action Observation OR

Action Observation Training OR

Action Observation Treatment OR

Action Observation Therapy OR

Action Observation Physical Training OR

Action Observation-Execution OR

Motor Observation OR

Movement Observation)

AND

(Rehabilitation (MESH) OR

Rehabilitation OR

Neurorehabilitation)

OR

(Motor function OR

Motor recovery OR

Motor learning OR

Motor activity OR

Motor ability OR

Functional recovery)

Filter HUMANS
